# Essential Medication in Cardiac Diseases1Read before the Medical Section, Buffalo Academy of Medicine, October 11, 1904.

**Published:** 1905-02

**Authors:** Eli H. Long

**Affiliations:** Professor of Materia Medica and Therapeutics, University of Buffalo


### Essential Medication in Cardiac Diseases.[1]

By ELI H. LONG, M. D.,
Professor of Materia Medica and Therapeutics, University of Buffalo.

IN THE treatment of diseases of the heart we are learning to disregard somewhat the precise pathologic distinctions and to look more to functional conditions for our indications. This is apt to be the attitude of the practitioner who, by years of experience, finds that rules of treatment based upon evident structural conditions are inefficient. The engaging study of heart pathology should not be discounted by this fact, but we should recognise that the heart in the living body cannot be studied and treated by itself, because of the complexity and the relations of the circulatory apparatus, of which it is the center. With this introductory thought, attention is first invited to a general division of cardiac cases into:

| | Nature. | Type. |
|---|---|---|
| Endocardial .......... | Inflammatory | Rheumatic |
| Myocardial ........... | Toxic | Pneumonic |
| | Degenerative | Sclerotic |

We may profitably also review the drugs upon which we place most reliance, noting briefly their physiologic action:

*Caffeine.*—Simply increases rapidity of action. A *kinetic* stimulant.

*Strychnine.*—Increases irritability of nerve centers. A *potential* stimulant.

*Digitalis Group.*—(*a*) Increase the force of systole, (*b*) contract the volume of a dilated heart and, (*c*) in full doses and in the absence of fever, tend to lessen rapidity and tonicity of contractions by stimulation of vagus centers; contract arterioles.

*Atropine.*—By paralysing the cardiac ends of the vagi, allows greater rapidity of action, with possible direct stimulation of the heart. Contracts arterioles.

*Nitrites.*—By paralysing vagus centers, allow marked increase of rapidity of action. Dilate arterioles.

*Aconite.*—By stimulating vagus centers, lessens the rapidity and force of cardiac contractions.

---

1. Read before the Medical Section, Buffalo Academy of Medicine, October 11, 1904.

This discussion will deal only with common forms of cardiac disease and with ordinary well-known remedies. We can well afford to ignore the numerous new but uncertain drugs, for the sake of a better acquaintance with the action and application of the few older drugs, the value of which has been proven by years of experience.

To commence with the usual beginning of cardiac valvular disease, let us suppose an ordinary case of acute rheumatic endocarditis in which a cardiac murmur has developed. We have in such a patient a heart that presents a deformity superadded to an acute inflammatory process, which is likely to lead to more or less disability. The question arises at once as to heart medication and particularly as to the use or disuse of digitalis. Keeping uppermost the question whether any medication is needed, we must view the condition both from the standpoint of the acute inflammation and in the interest of the future integrity of the heart's function. If we apply the principle of rest and removal of irritation, we shall employ measures that will lessen blood pressure, tension of inflamed tissues, and friction of blood current upon orifices and valves.

Rest of a certain kind will do this but not the rest that digitalis sometimes gives; for with sufficient dosage to lessen pulse-rate perceptibly the more powerful contractions induced, by increasing the intracardiac pressure and friction, will more than outweigh the advantage of a slight reduction of rate. If any drug is indicated it will be one that has the same slowing and relaxing vagus effect that digitalis has, without the muscular stimulant effect. Such a drug is aconite. The pharmacology of recent years has taught us that aconite is our purest inhibitory stimulant and that this action is its most definite one. We may therefore expect from this drug a lessening of rapidity and force of the heart's action, brought about by stimulation of the vagus centers. It is plainly indicated when the heart is disturbed and rapid, and the valvular disability is not so great as to forbid the reduction of blood pressure.

Viewing the question further in the interest of the future, it is plain that measures that will reduce the existing inflammation will thereby tend to lessen the resulting deformity of heart structures. On the other hand, it must be insisted that a drug of the digitalis group is capable of doing harm during acute endocarditis, by increasing blood pressure and intracardiac friction upon valves and orifices already in an irritated condition.

Dismissing the factor of endocarditis, the deformity next claims attention. Regarding this, the fact has become well known that the intensity or extent of a murmur does not furnish positive knowledge as to the seriousness of the lesion; and upon this fact we may base the statement that the presence of any murmur whatever does not furnish an indication for medication. A loud murmur may attend a very slight disability that requires little treatment, while serious leakages may produce murmurs of less intensity. The practice of some physicians of giving digitalis as a routine upon the discovery of a heart murmur is most unscientific and, as previously shown, may do much harm. At the same time I would add that even cases of mitral stenosis and aortic insufficiency, where digitalis is held by some to be contra-indicated, may at times present conditions of the general circulation that call for its temporary use.

Another factor that calls for discrimination is the pulse rate. Recognising that an increased pulse rate may mean a nervous heart, or a weakened circulation, or simply the reaction of the heart to meet greater demands, or the influence of a higher blood temperature, as in fever, we must conclude that simple rapidity of heart action does not furnish our best indication for medication. We must always observe the condition of the circulation as a whole in order to administer intelligent treatment. We will not always be able to form a satisfactory estimate of all factors at the first examination of a patient, particularly before we have a careful analysis of the urine to determine the presence or absence of toxic elements of faulty metabolism and elimination; but it is important to ascertain as early as possible the exact available heart energy expressed in functional power, and until this has been done only emergency medication should be employed.

As a first step, the work of the heart should be reduced to a minimum by enforcing a strict recumbent posture. In cases of uncertain or evidently diminished heart power, the patient should not be allowed to rise in bed for any purpose whatever, unless orthopnea requires the sitting posture temporarily. A few days will tell us whether the heart is able to maintain efficiently a minimum circulation, that is, on a level, with the antagonism of the force of gravity entirely eliminated. The rule to be followed from this point is this: as long as the circulation is maintained on a level sufficiently to allow of the organic functions in general being carried on normally, there is no need of heart stimulation. Practically, we find that the large majority of cases of recent endocarditis can be carried on to perfect compensation by attention to proper hygiene, bathing, graduated exercises and elimination, without resort to cardiac stimulants. I would note one exception to the rule, which is where the conditions favor early dilatation that will interfere with speedy compensation. The same general rule will apply as well to chronic older cases with broken compensation and to cases of heart strain.

The minority of cases, where the heart is incapable of doing even the minimum of work well, will soon present the picture of an efficient circulation. Let us regard these as emergency cases now, and as long as they require stimulation of the heart. It would be well also if we were to consider the digitalis group usually as emergency remedies, for it may well be argued that stimulation is needed only in emergency conditions.

The condition most evident in these cases is that of a disturbed balance between the arterial and venous sides of the circulation. Whether from disability or obstruction, the heart is unable to push the blood column onward with sufficient force to complete the return flow, and consequently we have a venous plethora. Let us note the reactionary result upon the heart, of this condition. The weakened circulation means first diminished oxygenation. An accumulation of blood in the venous side means cyanosis of the various organs concerned in general nutrition and in elimination. The lessened oxidation, faulty catabolism and deficient elimination mean a blood returned to the heart surcharged with effete matter and more or less toxic. The lungs return it only partially oxygenated, hence the coronaries receive a blood incapable of properly nourishing the heart and often positively toxic. The weak heart sends out the blood only to receive it again with its detrimental qualities increased.

Under these conditions, the disability of the heart must be progressive and the added symptoms of edema and dyspnea occur, with another condition less apparent but of greater import, that of cardiac dilatation. The meaning of the latter and its bearing upon treatment should receive greater attention in the study of heart conditions. Dilatation of the chambers of the heart is an accommodative, and, within certain limits, a normal variation; but in the kind of cases we are considering, we have a heart that is disabled but not compensated, where the stagnation of fluids in the periphery must be accompanied by over-filling of some of the internal reservoir-like spaces. The heart chambers and the aorta are called upon to accommodate the surplus. The fixed character of the disability leads to a point of actual strain of these organs, the result of which in young people is usually a stretching of the wall of the left ventricle and, to some extent, the walls of the other chambers of the heart. We should not fail to appreciate under what a great disadvantage the heart muscle is working when so stretched and with its cavity distended to such an extent that a considerable volume of blood must always remain after its contraction. It is very plain that the greater the distention the greater the disadvantage, and this must be regarded as a serious factor in itself apart from the valvular condition or pulse rate.

We have here three indications that must be met by the aid of medicines: (*a*) the weakness of the heart, (*b*) the peripheral stagnation, and (*c*) dilatation of the left ventricle. The vicious circle which has been established must be broken in two places,—by putting more force into the circulation, and by improving the quality of the blood through active elimination; and, it may be added, the latter is not less important than the former. Indeed, the beginning of our treatment with the patient in a recumbent posture may well be the administration of a hydragogue cathartic. Jalap, in form of the resin, or compound jalap powder, is one of the most valuable agents for this purpose, being nonirritating, very prompt, and free in its results. I have come to consider the giving of this drug for a considerable time, to the extent of producing from three to six fluid stools daily, as an essential part of the treatment in these cases.

Now as to the condition of the heart, what medicines will serve us best? Remembering that we have a heart that is responsive, as shown by the increased pulse rate, but one that is pulsating feebly because of disability and the disadvantage of some distention and dilatation of the ventricles, we can make quite precise application of our drugs. Strychnine does not meet the indications to any great degree, because of the uncertainty of its direct action upon the heart muscle. The most that we can expect of it is to keep the nerve centers and the cardiac muscle in a responsive condition. For this purpose it may well be employed. Caffeine acts chiefly by increasing the rapidity of the heart action. It does not make the pulsations more efficient, but its diuretic action may at times be valuable. Its disadvantages are, (*a*) that simply increasing rapidity and not efficiency may contribute to heart fatigue without any corresponding benefit and, (*b*) its tendency to produce insomnia.

Atropine allows greater rapidity of the heart by paralysing the vagus terminals and possibly stimulates the heart directly. It causes some contraction of the arterioles and tends to raise blood pressure, but after all is said, we must admit that atropine ranks as a second rate cardiac stimulant. Drugs of the digitalis group have so long been used with good effect in these cases, that no argument is needed to establish their value. We can only seek to better understand their action and to become more skilled in suiting them to precise indications. The diuretic value of these drugs will be referred to only in the statement that this action is largely dependent upon an increased blood pressure and is therefore secondary to, and accomplished by, their chief action. The two results to be sought in the action of these drugs is to bring the action of the heart up to the work required of it and to diminish the dilatation.

It might be questioned whether the direct stimulation of the weakened and fatigued heart muscle would be wise were it not that other advantages prevent an increase of fatigue. Digitalis in its moderate action contracts the volume of the heart by causing a more powerful contraction and slightly diminishes the pulse rate. Much has been said of the power of this drug to prolong diastole, and on that account its use has been discountenanced in certain lesions, particularly in aortic insufficiency, but we cannot forget that, given either in moderate or full dosage it causes a firmer ventricular contraction, which means a better emptying of the chamber, a longer systole and a better advantage given to the muscle, because the stretching is relieved and the bulk of blood to be propelled at each contraction is lessened. Systole may be prolonged as much as diastole with a counterbalancing of the disadvantage.

The power of digitalis to lessen the volume of the heart, thereby overcoming dilatation, must be regarded as a very important part of its action. It may even be asked whether the largest factor for good in its action is not the placing of the heart muscle at a better advantage by lessening the distention of its chambers? The result of the full action of the drug, including its vasoconstrictor action, is to raise blood pressure, the permanency of which depends very much upon our success in reducing the dilatation.

Yet our treatment not only aims at present improvement, but must have for its object complete compensation of the disability. This means a growth of the structure of the heart, which can only be brought about through securing to it a satisfactory degree of nutrition. Three points essential to this end are: adequate rest, a good blood, and a sustained arterial pressure which will supply to the coronaries their full quota for heart nutrition. All of these are aided by the judicious use of digitalis during the whole emergency period,—that is, until the heart is capable of performing its minimum work unaided.

As to the difference in action of the drugs of the digitalis group, our pharmacologists have not been able to give us any very important data. It has been believed that strophanthus causes less vasoconstriction than digitalis, but this seems now open to question, and we may say that digitalis still stands as the typical and chief one of the group, the others serving well as substitutes. A rule that relates to the use of these drugs in cardiac diseases in general is this,—that the more nearly normal the muscular tissue of the heart is, the more we may expect from their use, while the greater the degree of degeneration the less we may expect from their use and the less are they indicated.

Our case of valvular disease once fully compensated may remain so for life or, as so frequently happens, may again present with broken compensation due to various causes. But when this occurs we are introduced to a pathology that is essentially myocardial, for we have here a hypertrophied heart muscle unequal to its task, brought to this condition either by overloading or by interference with the nutrition. The case, therefore, approaches the degenerative type. But the foregoing remarks as to medication, in aid of again securing compensation, will apply to the degree that we have a heart muscle capable of improvement. We shall give no special consideration therefore to cases of this kind, but turn our attention to the degenerative type of disease.

We recognise well enough the important relation of general nutrition to heart nutrition, to know that the two are dependent upon each other and to appreciate the great importance of an efficient capillary circulation in every organ of the body, including the heart. When cardiac degeneration actually occurs, whether fibrous or fatty, we usually have some constitutional condition present, of which the cardiac change is only one evidence or result, for the statement will apply here that degenerative disease of the heart is usually secondary, the most common antecedents being a specific endarteritis, senile degeneration and faulty metabolism. We may add that it may likewise be secondary to the endocardial or valvular disease when compensation has been lost; so that the treatment of broken compensation may need to follow not only lines heretofore considered but those that will follow.

Let us now picture our typical case of myocardial disease. We have a heart hypertrophied to some degree, but now dilated, giving, it may be, a fairly strong impulse, but the circulation is not correspondingly efficient. The heart muscle has suffered some degeneration, in consequence of which we have irregularity. The rate of pulse is variable, rapid if the heart retains its normal irritability, slow if it does not. The peripheral circulation throughout is poor, primarily because of changes in the small arteries, to which the contribution of lessened cardiac forces is soon added. The hardening of the peripheral vessels occasions a certain resistance against which the heart is working. It is this added work that has doubtless led to the earlier hypertrophy of the heart and now, with the nutrition of that organ failing, the resistance still calls for extra work. Frequently no murmur is to be heard unless endocardial disease previously existed. In some cases, however, murmurs produced by either atheroma or dilatation of the aorta, or else caused by a relative insufficiency, may be heard.

The most essential points in this picture, in my view, are insufficient capillary circulation and degeneration of the heart structure. It would be interesting to study the relation of cause and effect in these two conditions did time permit, but it must suffice to say that, when the case is progressed to the point of showing serious cardiac symptoms, we have reached the result of years of disease in the circulation and that the time for most efficient treatment is forever past. The fate of the possessor of such a heart is well known to us and our efforts can only postpone the end. Present methods of treatment, however, do frequently prolong life for years, but it must be admitted that comparatively little of credit for this can be given to the administration of medicines. The importance of peripheral conditions, both circulatory and cellular, finds emphasis in the truly remarkable improvement that often follows the employment of the Schott bath and properly graduated exercise.

But we are chiefly concerned at this time with the influence of medication. In taking up the drugs which we are most likely to employ, we will consider, first, digitalis. Calling attention again to the two essential points, that of reduced peripheral circulation and that of heart degeneration, we find two serious objections to the use of digitalis. First, its vasoconstrictor action will tend to still further reduce the capillary circulation and increase the peripheral resistance, which will add to the work of the heart. Second, the muscular tissue of the heart has been reduced in quantity, thus presenting less tissue for the characteristic and best action of the drug. It is commonly held that this drug is contraindicated in fatty degeneration, but it is probably better to state that the less of good muscular tissue remaining in the heart, the less can we expect any good result from a drug of this class.

The two objections given might be sufficient to forbid its use were it not for two very decided indications; one is to reduce dilatation and the other to secure a better nutrition of the heart by raising arterial pressure. Much as we say about the influence of trophic nerves of the heart, the trophic influence that appeals most to our knowledge and responds most to our efforts comprises the coronary arteries well filled with blood of good quality, and this is an essential condition of improvement. Active elimination in order to improvement of the blood, is here again called for as in the acute cases. The reduction of dilatation, it seems to me, is so important a matter as to call for special effort, provided there is still enough muscle tissue left to respond to digitalis. The heart is thus given the best possible advantage in its action and a great contribution will be made toward securing its better nutrition. The first objection, that of its peripheral influence, must be overcome, in case the drug is administered, by the combined use of vasodilators and by the employment of baths, massage, and exercise. These are the cases in which we must carefully weigh our indications and contraindications in arriving at a course of treatment.

Before passing to the consideration of other drugs, permit me to call attention to another factor which is not always of minor importance,—the pulse rate. It cannot be stated that we have a characteristic pulse rate of the degenerated heart; neither can we say that the slow pulse which is frequently seen is due to excessive inhibition. It may be due to a diminished power of reaction in the heart structure. It would seem reasonable to regard rapidity of the pulse as an index of the reactive power of the heart and to be encouraged within normal limits. A very slow pulse would present another objection to digitalis; however, I recall a case where a slow, irregular pulse became more rapid and regular under digitalis, doubtless through improvement of heart nutrition and its power of reaction. On the other hand, atropine would be well suited to a case presenting this feature. By stimulating directly the heart muscle and lessening the opposing inhibitory influence, it might give us a very decided advantage. The group of nitrites, well represented by glonoin and sodium nitrite, have a similar action in that they paralyse INHIBITION, but they are superior in their peripheral effect in that they allow a free capillary circulation by dilating the arterioles. These drugs have not been proven to be direct heart stimulants. The acceleration of pulse is due to depression of the vagus and the vasodilator effect to paralysis of the muscular coat of the arterioles. Inasmuch as their action is essentially a depressant one, the propriety of their continuous use may be questioned, but as an aid to an overburdened and poorly nourished heart, they will serve us by removing peripheral resistance and permitting a freer capillary circulation. With or without digitalis, their temporary use is often of decided advantage; but, as in the case of digitalis, they would better be regarded as emergency drugs and permanency of their effects maintained by nonmedical measures. The main object to be sought always is not stimulation of the heart, but an improvement in its nutrition.

1335 MAIN STREET.

